# Support for the elevational Rapoport's rule among seed plants in Nepal depends on biogeographical affinities and boundary effects

**DOI:** 10.1002/ece3.2473

**Published:** 2016-09-21

**Authors:** Jianmeng Feng, Xiaokang Hu, Jie Wang, Yanmei Wang

**Affiliations:** ^1^ Department of Life Science and Chemistry Dali University Dali China

**Keywords:** biogeographical affinities, elevational gradient, elevational range size, hard boundary effects, Nepal, species diversity, the elevational Rapoport's rule

## Abstract

As one of the most important hypotheses on biogeographical distribution, Rapoport's rule has attracted attention around the world. However, it is unclear whether the applicability of the elevational Rapoport's Rule differs between organisms from different biogeographical regions. We used Stevens’ method, which uses species diversity and the averaged range sizes of all species within each (100 m) elevational band to explore diversity‐elevation, range‐elevation, and diversity‐range relationships. We compared support for the elevational Rapoport's rule between tropical and temperate species of seed plants in Nepal. Neither tropical nor temperate species supported the predictions of the elevational Rapoport's rule along the elevation gradient of 100–6,000 m a.s.l. for any of the studied relationships. However, along the smaller 1,000–5,000 m a.s.l. gradient (4,300 m a.s.l. for range‐elevation relationships) which is thought to be less influenced by boundary effects, we observed consistent support for the rule by tropical species, although temperate species did not show consistent support. The degree of support for the elevational Rapoport's rule may not only be influenced by hard boundary effects, but also by the biogeographical affinities of the focal taxa. With ongoing global warming and increasing variability of temperature in high‐elevation regions, tropical taxa may shift upward into higher elevations and expand their elevational ranges, causing the loss of temperate taxa diversity. Relevant studies on the elevational Rapoport's rule with regard to biogeographical affinities may be a promising avenue to further our understanding of this rule.

## 1 Introduction

Biodiversity patterns are among the hottest topics in ecology and biogeography. However, spatial patterns of species’ range sizes along geographical gradients, which to a certain extent underlie the biodiversity patterns, have received much attention in the past decades (Connolly, 2009; Gaston & Chown, 1999; Sizling, Storch, & Keil, 2009; Taylor & Gaines, 1999; Tomasovych, Jablonski, Berke, Krug, & Valentine, 2015; Tomasovych et al., 2016; Vazquez & Stevens, 2004), compared with those on biodiversity patterns it seems to attract less attention (McCain & Knight, 2013). The elevational Rapoport's rule (ERR), which predicts trends of increased elevational ranges with the increase in elevation (Stevens, 1992), has received much attention in recent decades (e.g., Bhattarai & Vetaas, 2006; Fu, Wu, Wang, Lei, & Chen, 2004; McCain & Knight, 2013; Patterson, Pacheco, & Solari, 1996; Sanders, 2002) and is still catching attention from ecologists and biogeographers around the world (e.g., Garcia‐Rosello et al., 2015; Rohner et al., 2015; Sheldon, Leache, & Cruz, 2015; Tomasovych et al., 2015). Two issues may be responsible for this attention. First, considerable controversies about ERR remain, as there is a high degree of variability in support for this hypothesis. For example, some studies found strong support for ERR (e.g., Chatzaki, Lymberakis, Markakis, & Mylonas, 2005; Fleishman, Austin, & Weiss, 1998; Gaston & Chown, 1999; Hausdorf, 2006; Patterson et al., 1996; Ribas & Schoereder, 2006; Sanders, 2002), whereas others found little or no support (Bhattarai & Vetaas, 2006; Fu et al., 2004; Nathan & Werner, 1999; Patterson et al., 1996; Rahbek, 1997; Ribas & Schoereder, 2006). Second, elevational range shift caused by global climate change may increase the risk of extinction for small‐ranged species (La Sorte & Jetz, 2010; McCain & Colwell, 2011; Sekercioglu, Schneider, Fay, & Loarie, 2008). Thus, more studies on ERR may not only help to deepen our understanding of ERR, but also be helpful to conserve biodiversity, especially in small‐ranged species.

The core prediction of ERR is a positive relationship between range size and elevation (Stevens, 1992). However, the patterns of elevational range size may be taxon‐specific (McCain & Knight, 2013), which suggests that range‐elevation relationships may depend on the adaptation of ecophysiological traits to climatic or environmental factors along elevation gradients. Due to strong associations between ecophysiological traits of taxa and their biogeographical affinities (e.g., compared with tropical species, temperate species may be more resistant to cold weather) (Wilson, 1975), biogeographical affinities may be linked with elevational range sizes and their elevational trends (e.g., compared with tropical taxa, temperate taxa show broader elevational range sizes, as the latter may have experienced higher variability of environmental factors in their evolutionary or biogeographical history) (Oommen & Shanker, 2005; Wang, Tang, & Fang, 2007). However, little attention has been paid to range‐elevation relationships with regard to the influence of biogeographical affinities.

Another prediction of ERR is that species diversity decreases with increasing elevation. However, this has not been consistently observed. Elevational trends in diversity other than monotonic decreasing ones, that is, unimodal trends, have also been observed worldwide, suggesting that further investigations of this hypothesis are needed. There are strong linkages between ecophysiological traits and biogeographical affinities (e.g., taxa with tropical affinities may be prone to inhabit warm climates) (Wilson, 1975), which may imply that taxa with different biogeographical affinities may show differentiated adaptation to environmental factors, including elevation. Species with different biogeographical affinities may show different elevational patterns of richness, resulting in varying support for the richness–elevation hypothesis of ERR. To date, few studies have tested this assumption (e.g., Li & Feng, 2015; Oommen & Shanker, 2005).

The third prediction of ERR is an inverse relationship between species richness and elevational range (Stevens, 1989, 1992). This hypothesis has not been tested extensively. However, when predictors of climatic variability or source–sink dynamics show weaker influences than expected, the hypothesis remains in question (Kerr, 1999; Ribas & Schoereder, 2006).

The hypothesis of hard boundary effects predicts that the unimodal patterns of species diversity on elevational gradients are caused by the increasing overlapping of species ranges toward the centers of the elevational gradient, as species range is bounded by upper and lower limits (boundaries) of the elevational gradient (Colwell & Hurtt, 1994). This hypothesis also predicts that species range increases toward the centers of the elevational gradient. Overall, this hypothesis contradicts the ERR. Since 1994, when this hypothesis was proposed, it has remained controversial (Colwell, Rahbek, & Gotelli, 2005; Hawkins, Diniz‐Filho, & Weis, 2005; Herzog, Kessler, & Bach, 2013; Kluge, Kessler, & Dunn, 2006).

Using an online dataset of seed plants in Nepal, this study tested the following hypotheses: (1) the range‐elevation, diversity‐elevation, and range‐diversity relationships vary with biogeographical affinities and (2) reduction in hard boundary effects may enhance support for ERR, which may also depend on the biogeographical affinities.

## 2 Materials and Methods

### 2.1 Area description

**Figure 1:**
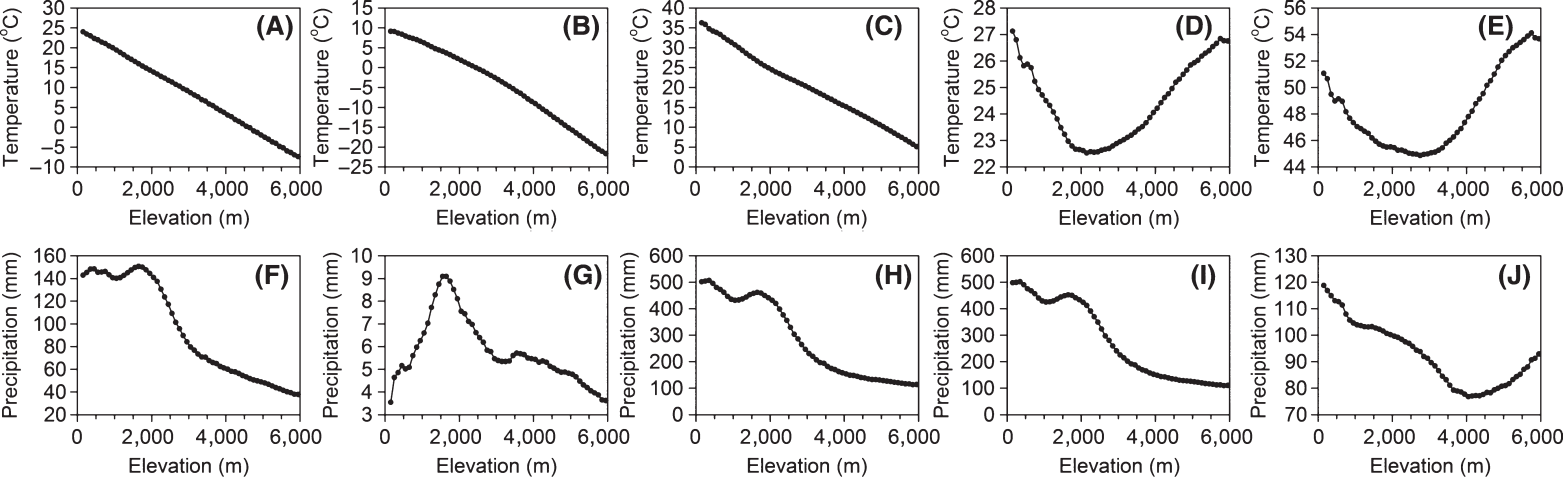
Temperature seasonality‐elevation (A), min temperature of the coldest month‐elevation (B), max temperature of the warmest month‐elevation (C), annual temperature range‐elevation (D), annual mean temperature‐elevation (E), precipitation seasonality‐elevation (F), precipitation of the driest month‐elevation (G), precipitation of the wettest month‐elevation (H), annual precipitation range‐elevation (I), and mean monthly precipitation‐elevation (J). All of the data were downloaded from the Worldclim (http://www.worldclim.org/) (Hijmans, Cameron, Parra, Jones, & Jarvis, 2005)

Our study area was the whole country of Nepal (80°04′–88°12′E, 26°22′–30°27′N), with a total area of 147,181 km^2^. Topographically, Nepal is characterized by extreme elevational variation; for example, in some locations, elevation changes from about 60 to 8,848 m a.s.l. in just 150 km of horizontal distance (Hagen, 1969; Manandhar, 1999). The area at each elevation decreases until around 3,750 m a.s.l. and then shows a slight increasing trend. Mean monthly precipitation has two peaks along the elevational gradient, at about 300–400 and 1,600–1,700 m a.s.l., and decreases with further increases in elevation; Mean annual temperature linearly decreases with elevation, from 24.1°C around 100 m a.s.l and declining by 0.55°C per 100 m elevation (Fig. 1). Temperature seasonality, annual temperature range, and precipitation seasonality decrease and then increase along the elevational gradient from 100 to 6,000 m a.s.l as well as within the smaller range of 1,000–5,000 m a.s.l., showing open‐upward‐parabola patterns (Fig. 1). Annual precipitation range (precipitation of the wettest month precipitation of the driest month) on elevation showed patterns similar to that of mean monthly precipitation (Fig. 1). Cloud formation is mostly around 2,000 m a.s.l. (Bhattarai & Vetaas, 2003) in the lesser Himalayas, whereas in the greater Himalayas, it is normally between 2,500 and 3,200 m a.s.l. (Bhattarai & Vetaas, 2003; Dobremez, 1976). Consistent with the elevational climatic patterns, with the increase of elevation one can observe tropical/subtropical, temperate, subalpine, and alpine vegetation zones, respectively (Bhattarai & Vetaas, 2003; Dobremez, 1976).

### 2.2 Plant data sources

We obtained plant information from the online version of the Annotated Checklist of the Flowering Plants of Nepal (http://www.efloras.org/, accessed on 1 December 2014). This dataset gave us family, genus, and species identified across the country, and the minimum and maximum elevations of each. We considered a small number of varieties and subspecies as separate species for this study. In all, there were 4,914 species, representing 222 families and 1,426 genera.

### 2.3 Subdivision of the study area into elevational bands

We divided the study area into 59 elevational bands, each with 100 m altitude change, from 100 to 6,000 m a.s.l. (100–200 m a.s.l., 200–300 m a.s.l., and so on). Grytnes and Vetaas (2002) suggested that the interpolation method, used in the present study, may underestimate species diversity at the gradient extremes. In addition, hard boundary effects may significantly reduce ranges and species diversity around gradient extremes (Colwell & Hurtt, 1994). Hard boundary effects on plant diversity in Nepal are mostly observed below 1,000 m a.s.l. and above 5,000 m a.s.l. (Grytnes & Vetaas, 2002). As both of these biases may reduce the support for ERR, we focused our attention on the elevational gradient from 1,000 to 5,000 m a.s.l., which we assumed strongly reduced hard boundary effects. This method was adopted by Vetaas and Grytnes (2002) and led to robust conclusions. We surmised that compared with the support for the ERR observed on the full elevational gradient from 100 to 6,000 m a.s.l., the support within the smaller elevational gradient would be stronger.

### 2.4 Biogeographical affinities

Following Harrison and Grace (2007) and Wang, Fang, Tang, and Lin (2011), we assigned each species within a genus the same biogeographical affinity, using a classification system of biogeographical affinities (Wu, 1991). This system determines biogeographical affinity of species according to their biogeographical history, fossil records, and modern distribution, especially the modern distribution centers of the species (Wu, 1991). The distribution centers located in pantropic regions, including tropical Asia and tropical America, old world tropic regions, tropical Asia to tropical Australia, tropical Asia to tropical Africa, and tropical Asia were all considered tropical species. Those from north temperate regions, including east and north Asia, America, old world temperate regions, temperate Asia, Mediterranean, west to central Asia, central Asia, and east Asia were considered temperate species. Species and genera that span from tropics to temperate regions and have no obvious distribution centers were considered cosmopolitan species or genera. This method was adopted in a variety of study cases (e.g., Li & Feng, 2015; Qian, 1998; Qian et al., 2006; Wang, You, & Feng, 2015; Zhu, 2012). We assigned biogeographical affinities to 4,699 species, including 1,972 tropical species (40.5%), 2,142 temperate species (44.0%), and 585 cosmopolitan species (12.0%). Subsequent analyses considered only tropical and temperate species.

### 2.5 Variables considered

We focused on elevational patterns of the following variables: (1) elevational range sizes of tropical species (ERTRS); (2) elevational range sizes of temperate species (ERTES); (3) tropical species diversity (TRSD); and (4) temperate species diversity (TESD). To correct for the influence of area on elevational patterns of plant diversity, we used area‐adjusted species diversity (species density) instead of species richness, as follows:

D=S/ln(A)

where *D* is the species density for each elevational band, *S* is the number of species in the elevational band, and *A* is the area of the elevational band.

### 2.6 Diversity‐elevation and biogeographical affinities

We counted the number of species which occurred in each elevational band and generated the elevational patterns of species diversity (TRSD and TESD), for two ranges, 100–6,000 and 1,000–5,000 m a.s.l. In consideration of unimodal patterns of species density along elevational gradients, we used general additive models (GAM) with Gaussian function of variance to determine the trends of the response curve instead of using linear correlation analysis. In this method, a cubic smooth spline (Hastie & Tibshirani, 1990) was used to evaluate the significance of specific trend for diversity‐elevation relationships, as well as for range‐elevation and diversity‐range relationships. These analyses were carried out using R (https://www.r-project.org/).

### 2.7 Range‐elevation and biogeographical affinities

We used Stevens’ (1992) method and GAM to investigate elevational patterns of ERTRS and ERTES within 100–6,000 and 1,000–5,000 m a.s.l.

### 2.8 Diversity‐range and biogeographical affinities

We used Stevens’ method and GAM to explore diversity‐range relationships for both tropical and temperate species within both elevational ranges, 100–6,000 and 1,000–5,000 m a.s.l. It must be noted that the three approaches used to test the three predictions are not really independent tests, and diversity‐range relationships follow from diversity‐elevation and range‐elevation relationships.

## 3 Results

### 3.1 Range‐elevation and biogeographical affinities

**Figure 2:**
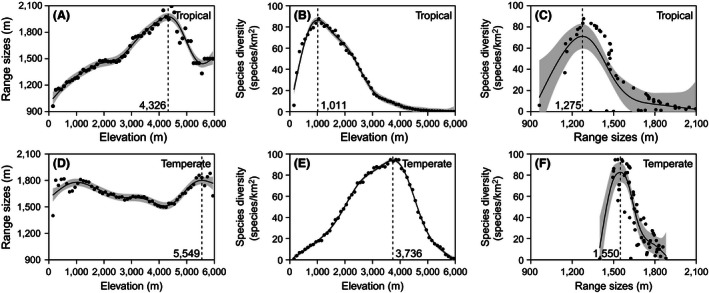
Range size‐elevation (A), species diversity‐elevation (B), and diversity‐range sizes (C) relationships for tropical and temperate species from 100 to 6,000 m a.s.l

**Figure 3:**
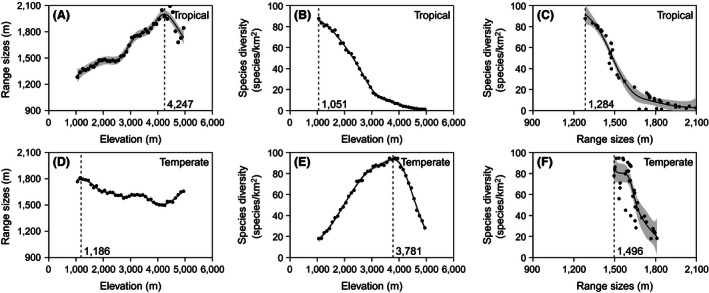
Range size‐elevation (A), species diversity‐elevation (B), and diversity‐range sizes (C) relationships for tropical and temperate species from 1,000 to 5,000 m a.s.l

General additive models showed that with increased elevation from 100 to 6,000 m a.s.l., ERTRS significantly increased to about 4,300 m a.s.l., decreased until about 5,500 m a.s.l., and then showed a slightly increasing trend. From 1,000 to 5,000 m a.s.l., ERTRS had a unimodal pattern, with maximum diversity at about 4,300 m a.s.l. (Figs 2 and 3). The GAM models also showed that with increased elevation from 100 to 6,000 m a.s.l., ERTES increased with elevation until 1,150 m a.s.l., decreased until about 4,150 m a.s.l., increased again until about 5,450 m a.s.l., and then decreased. (*p *< .001) (Fig. 2). From 1,000 to 5,000 m a.s.l., ERTES decreased until about 4,150 m a.s.l. and then increased (Fig. 3).

### 3.2 Diversity‐elevation patterns and biogeographical affinities

General additive models showed that with the increase of elevation from 100 to 6,000 m a.s.l., both TRSD and TESD increased to some point and then decreased. TRSD peaked at about 1,000 m a.s.l., while the maximum TESD was at about 3,750 m a.sl. (Fig. 2). GAM models also showed within the range 1,000 to 5,000 m a.s.l., TRSD consistently decreased with increasing elevation but TESD still showed a unimodal pattern (Fig. 3).

### 3.3 Diversity‐range and biogeographical affinities

GAM models showed that from 100 to 6,000 m a.s.l., there were no linear negative relationships between species density and range sizes for either tropical or temperate species (Fig. 2). However, from 1,000 to 5,000 m a.s.l., there were negative relationships between species density and range size for both tropical and temperate species (Fig. 3).

## 4 Discussion

We observed strong support for the range‐elevation relationships predicted by ERR in tropical species from 1,000 to 4,300 m a.s.l., where their elevational ranges linearly increased. However, from 4,300 to 5,000 m a.s.l., there was a trend of decreasing ERTRS, which may imply that although we truncated the higher end of the elevational gradient by 1,000 m, it was not enough to eliminate boundary effects such as environmental or climatic conditions. These effects may strongly constrain the distribution of tropical species and thus reduce their elevational ranges, as tropical species may not have strong resistance to the harsh climate at very high elevations (Wilson, 1975). It may imply that if boundary effects are eliminated, the elevational patterns of the tropical ranges may support the range‐elevation relationship predicted by ERR. However, there was a decreasing trend of ERTES in most of the elevational gradient from 1,000 to 4,300 or 5,000 m a.s.l. In this case, the reduction or even the elimination of boundary effects did not result in obvious support for the range‐elevation relationship predicted by ERR.

McCain (2009) observed that bats, mostly observed in subtropical or tropical regions, showed a stronger positive trend of elevational range sizes across latitudes than rodents, numerous in arctic regions, and possessing wider adaptability. This is somewhat analogous to the varying support for the range‐elevation relationship predicted by ERR by tropical and temperate species when hard boundary effects were accounted for in the present study. Compared with temperate taxa, tropical taxa may be more responsive to climatic variability and thus increase their elevational range sizes to adapt to an increase in climatic variability (McCain, 2009). In contrast, temperate taxa may have experienced wider adaptability to climatic variability in their evolutionary history and may therefore be less responsive to the increasing variability at higher elevations, contradicting the ERR. It must be noted that none of annual variability of climatic factors (e.g., temperature seasonality, precipitation seasonality, annual temperature range, and annual precipitation range) shows consistently increasing trends with the increase of elevation (Fig. 1). Therefore, we propose that increasing variability of daily variation in temperatures with elevation (e.g., Ghalambor, Huey, Martin, Tewksbury, & Wang, 2006; Porter, Sabo, Tracy, Reichman, & Ramankutty, 2002) may cause the increasing trends in ERTRS, although we were unable to test this due to a lack of reliable datasets. It may be that when hard boundary effects are accounted for, the support for the range‐elevation relationship predicted by ERR may primarily depend on biogeographical affinities of the focal taxa.

Our results showed that from 100 to 6,000 m a.s.l., both TRSD and TESD showed unimodal patterns, suggesting that hard boundary effects may strongly shape the elevational patterns of species density (Colwell et al., 2005; Grytnes & Vetaas, 2002; Lee, Chun, Song, & Cho, 2013). This may have reduced the strength of the support for the diversity‐elevation relationship predicted by ERR. However, from 1,000 to 5,000 m a.s.l., there was a consistent decrease in TRSD and unimodal patterns of TESD along the elevation gradient. This may be due to strong rescue effects in tropical species and weak rescue effects in temperate species. However, we cannot deny that decreases in water‐related variables with increased elevation may strongly shape elevational diversity patterns of tropical taxa, or that decreasing energy availability with elevation mainly influences diversity patterns of temperate taxa in quadratic terms (Li & Feng, 2015). Taken together, we can reasonably infer that the support for the diversity‐elevation relationship predicted by ERR may be modified not only by hard boundary effects but also by the biogeographical affinities of the taxa. It therefore may, to some extent, explain why even when the hard boundary effects were reduced, the diversity‐elevation relationships predicted by the ERR were not supported (Vetaas & Grytnes, 2002).

In the present study, for both tropical and temperate species, we did not observe consistent support for the three relationships predicted by ERR over the broad range from 100 to 6,000 m a.s.l. However, when hard boundary effects were accounted for, we observed consistent support for all three predicted relationships by tropical species. Temperate species did not show consistent support for the predicted relationships. This may suggest that when hard boundary effects are accounted for, support for ERR may be primarily biogeographical‐affinity specific. In sum, studies on the elevational form of Rapoport's rule with regard to biogeographical affinities may be a promising avenue to further our understanding of this rule, as suggested by McCain and Knight (2013), although the influence of hard boundary effects cannot be overlooked.

Since c. 1901 and 1961, the increasing trends of the rise of annual mean temperature with elevation have been observed in most of regional groups of meteorological stations around the world (Ohmura, 2012). With global warming, there is an increasing rate of warming with altitude, propelled by a rapid increase in daily minimum temperature (Diaz & Bradley, 1997), which may result in strong trends of upward shifting of tropical taxa from lower elevational or latitudinal regions (Anderson, Storlie, Shoo, Pearson, & Williams, 2013; Freeman & Freeman, 2014; Hickler et al., 2012; Thuiller, Lavorel, Araujo, Sykes, & Prentice, 2005; Vegas‐Vilarrubia, Nogue, & Rull, 2012). The present study suggests that tropical species seem to be sensitive to climatic variability on an elevational gradient, showing wider elevational range sizes with the increase of climatic variability (Chen et al., 2011) when hard boundary effects were reduced. These findings together may suggest that with ongoing global climate change, tropical taxa in lower elevational regions may shift upward and expand their elevational ranges into higher elevational regions already occupied by temperate taxa, causing temperate taxa contract habitats or lose habitats entirely, and hence cause the loss of temperate taxa diversity (Franco et al., 2006; Vegas‐Vilarrubia et al., 2012). This may pose a tough challenge to biodiversity conservation, especially for temperate taxa in higher elevational regions.

## Conflict of Interest

None declared.
